# Supplementary material for: DHX9-dependent recruitment of BRCA1 to RNA promotes DNA end resection in homologous recombination
Source: Nat Commun. 2021 Jul 5;12:4126. doi: 10.1038/s41467-021-24341-z (PMC8257769; doi:10.1038/s41467-021-24341-z)
Supplement: Supplementary file 1 — Supplementary Information [file 41467_2021_24341_MOESM1_ESM.pdf]

# Supplementary Figure 1

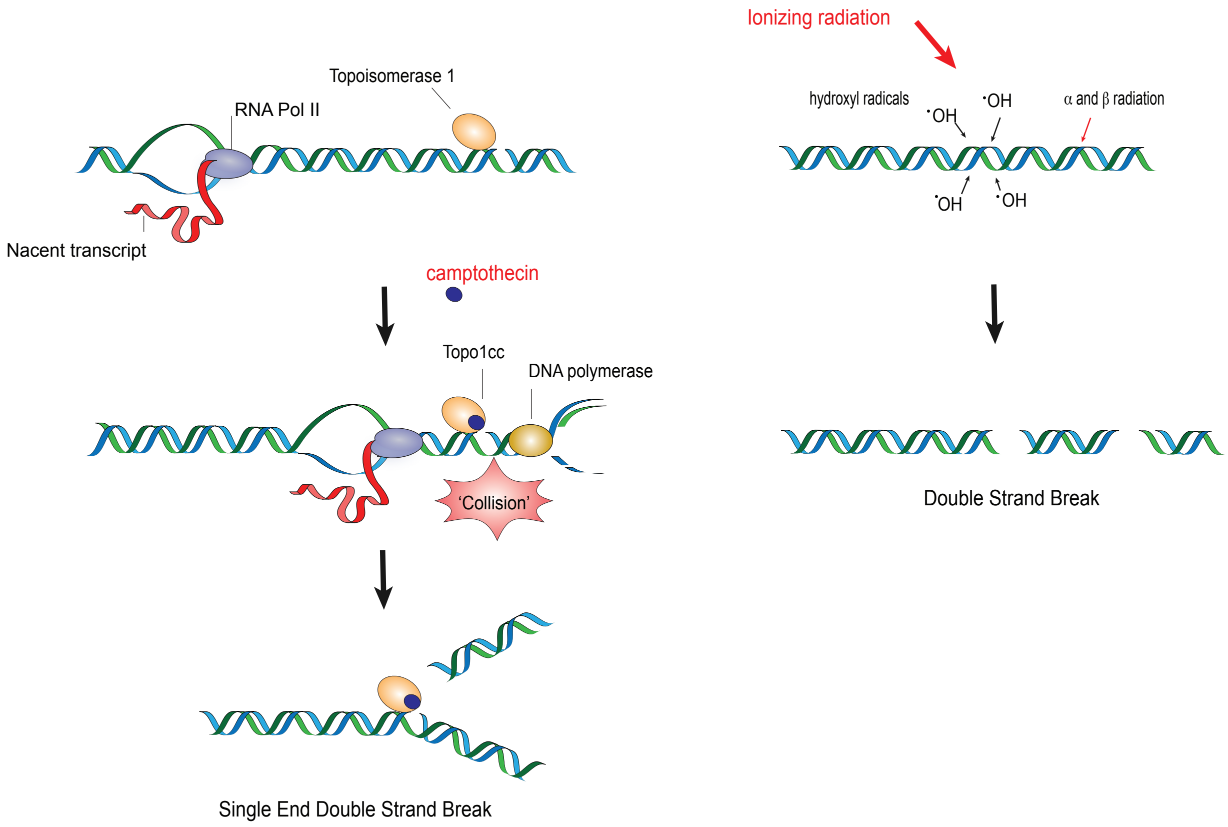

**Supplementary Fig 1.** Generation of DSB by camptothecin and ionizing radiation. *Left panel:* Treatment of cells with camptothecin inhibits Topisomerase I (topo 1), trapping it as a cleaved single stranded DNA intermediate in which topo 1 is covalently attached to the 5' broken end. These lesions, referred to as Topo I cleaved complexes (Topo 1cc), are enriched in highly transcribed loci and may lead to stalling of RNA Polymerase II near the break site. In replicating cells this also leads to the obstruction, stalling and collapse of a replication fork, causing the generation of a single-ended double strand break. *Right Panel*: Ionizing radiation emits alpha and beta particles that directly break the sugar-phosphate backbone of DNA causing the generation of DSB and SSB. Ionization of water molecules leads to the generation of hydroxyl radicals which also directly attack the sugar-phosphate backbone of DNA to generate DNA breaks. Source data are provided as a Source Data file.

Supplementary Figure 2

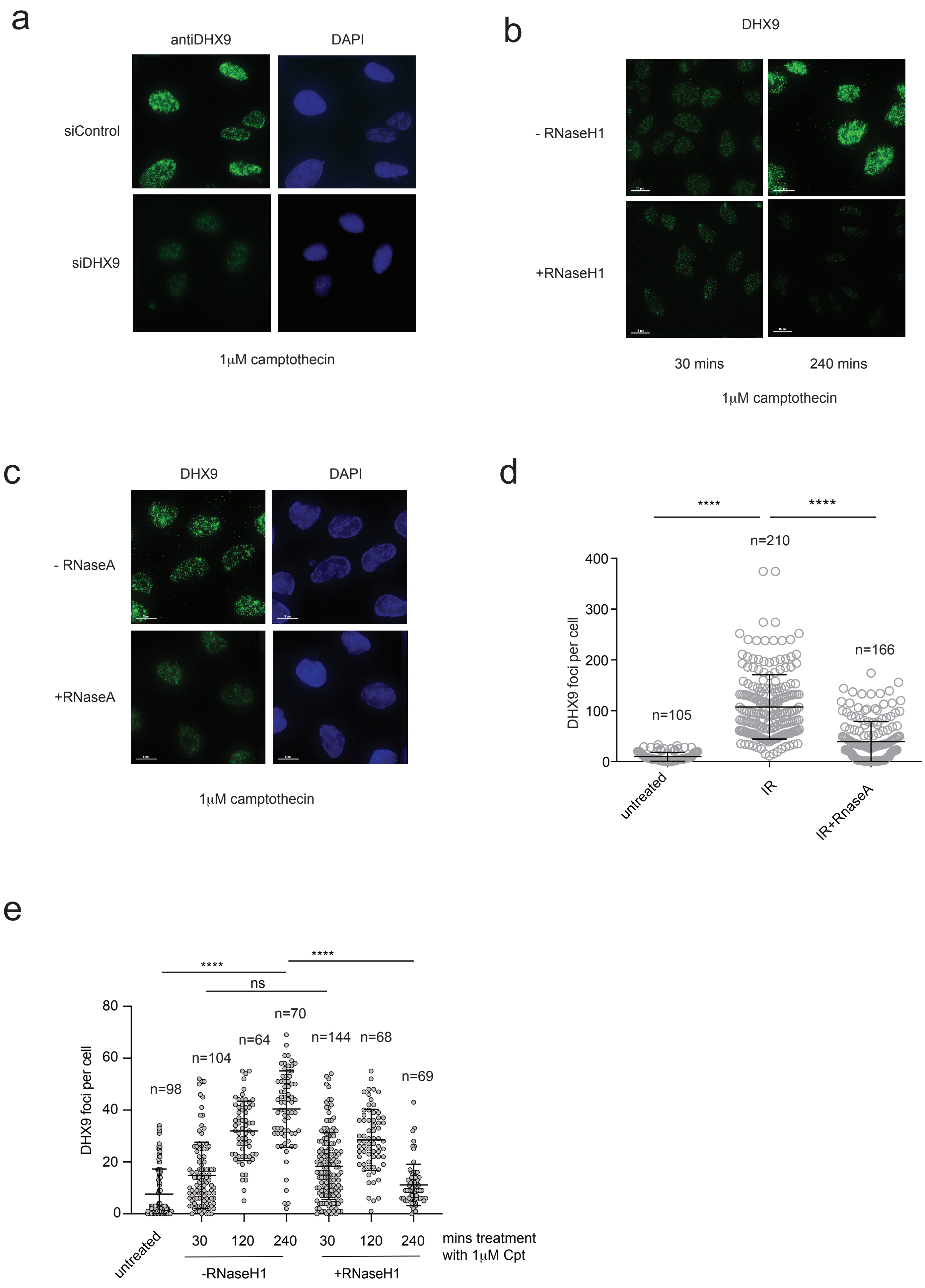

**Supplementary Fig 2.** (**a**) Fluorescence images showing that camptothecin induced DHX9 nuclear foci in U2OS cells are detected by antibody to DHX9 and are absent in cells depleted of DHX9 using siRNA. (**b**) Images showing that DHX9 nuclear foci, induced by treating cells with camptothecin, are disassembled by treating cells with RNaseA. (**c**) Graph showing that DHX9 foci, induced by treating cells with ionizing radiation, are disassembled by treating cells with RNaseA. Mean and standard deviation are shown. (**d**) Fluorescence images showing that early forming faint DHX9 foci are resistant to treatment with RNaseH1 while later forming intense foci are sensitive to RNaseH1 treatment. (**e**) Quantification of DHX9 foci induced in cells treated with 1 μM camptothecin for different times. The sensitivity of these foci to treatment with RNaseH1 is also presented. Quantification of *n* cells (as indicated) from 3 pooled biologically independent experiments were performed in (d) and (e). Statistical significance was determined using one way ANOVA with Tukey's post hoc test (****$p < 0.0001$, ns=not significant). Mean and error bars indicating one standard deviation are also indicated. Source data are provided as a Source Data file.

Supplementary Figure 3

a

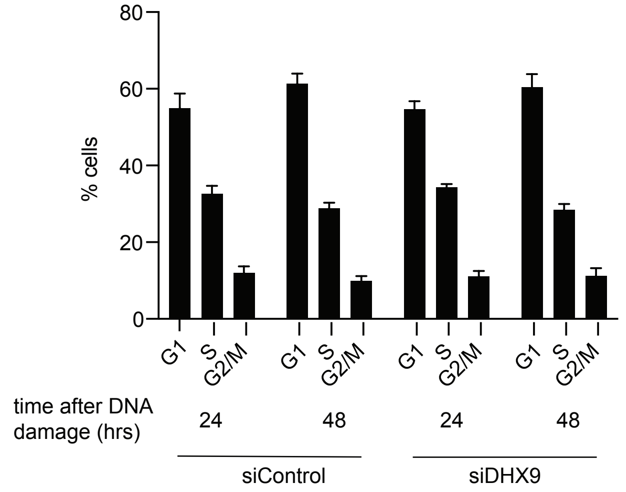


| Replicate | siControl 24 hrs 1 | siControl 24 hrs 2 | siControl 24 hrs 3 | siControl 48 hrs 1 | siControl 48 hrs 2 | siControl 48 hrs 3 | siDHX9 24 hrs 1 | siDHX9 24 hrs 2 | siDHX9 24 hrs 3 | siDHX9 48 hrs 1 | siDHX9 48 hrs 2 | siDHX9 48 hrs 3 |
|---|---|---|---|---|---|---|---|---|---|---|---|---|
| **G1** | 58.6 | 55.3 | 50.9 | 62 | 58.4 | 63.6 | 54 | 57 | 52.8 | 64 | 60 | 57 |
| **S** | 32.4 | 30.5 | 34.8 | 28 | 30.5 | 28 | 34.2 | 33.5 | 35.2 | 26.9 | 28.4 | 30 |
| **G2/M** | 10 | 12.7 | 13.2 | 10 | 11.1 | 8.4 | 11.8 | 9.5 | 12 | 9.1 | 11.6 | 13 |

b

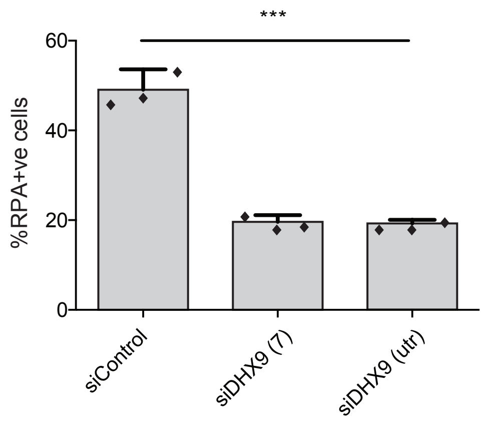


c

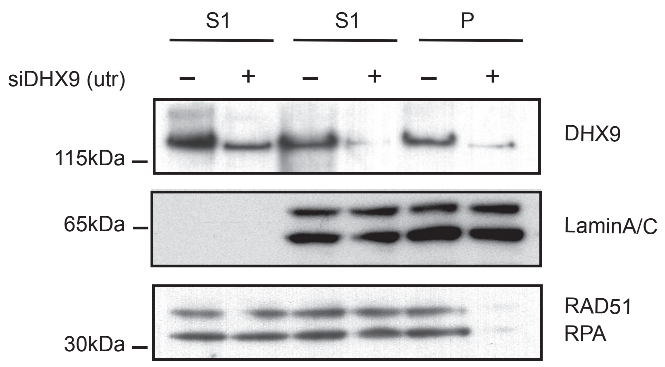


d

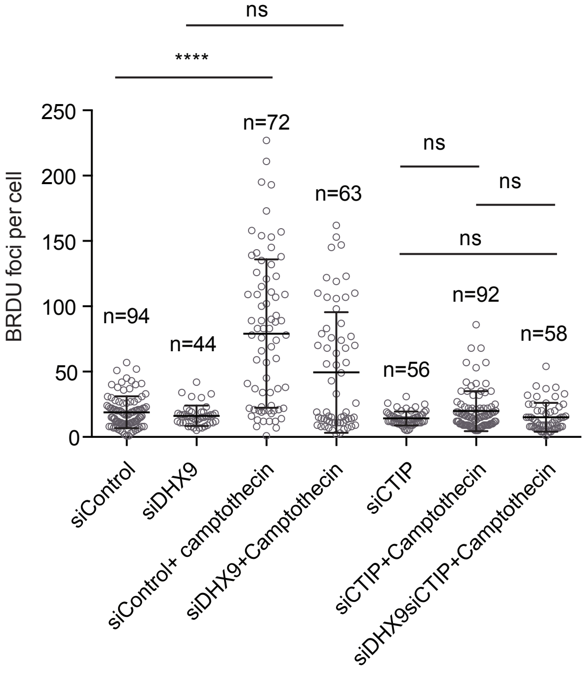

**Supplementary Fig 3.** (**a**) Graph showing percentage of cells in different phases of the cell cycle 24 and 48 hours after treatment with 1 μM camptothecin. Cells depleted of DHX9 using a specific siRNA and control cells treated with a non-specific siControl RNA are shown. Cell cycle phase was determined by FACS analysis of cells pulsed with BrdU and stained with propidium iodide as described previously[6]. Table below shows primary data for three biologically independent experiments (**b**) Graph showing that knockdown of DHX9 with two different siRNAs impairs the recruitment of RPA to chromatin. Percentage of cells staining with antibody raised against RPA and quantified using FACS for three biologically independent experiments. (**c**) Western blot showing that incorporation of RAD51 and RPA into the chromatin pellet (P) is reduced in cells knocked down for DHX9 using siDHX9(utr). Cells were fractionated as described in materials and methods into cytoplasm (S1), nuclear soluble (S2) and insoluble pellet (P). Lamin A/C is shown to confirm separation of nuclear and cytoplasmic fractions. (**d**) Graph showing that depletion of DHX9 impairs the generation of single stranded DNA by DNA end-resection in cells treated with 1μM camptothecin. Cells knocked down for the DNA resection protein CTIP are also shown as a positive control. Single stranded DNA was quantified as the number of BrdU foci staining with specific antibody in fixed cells and detected using fluorescence imaging. For all graphs, quantification of *n* cells (as indicated) from 3 pooled biologically independent experiments were performed. Statistical significance was determined using one way ANOVA with Tukey's post hoc test (****$p < 0.0001$, ns = not significant). Mean and error bars indicating one standard deviation are also indicated. Source data are provided as a Source Data file.

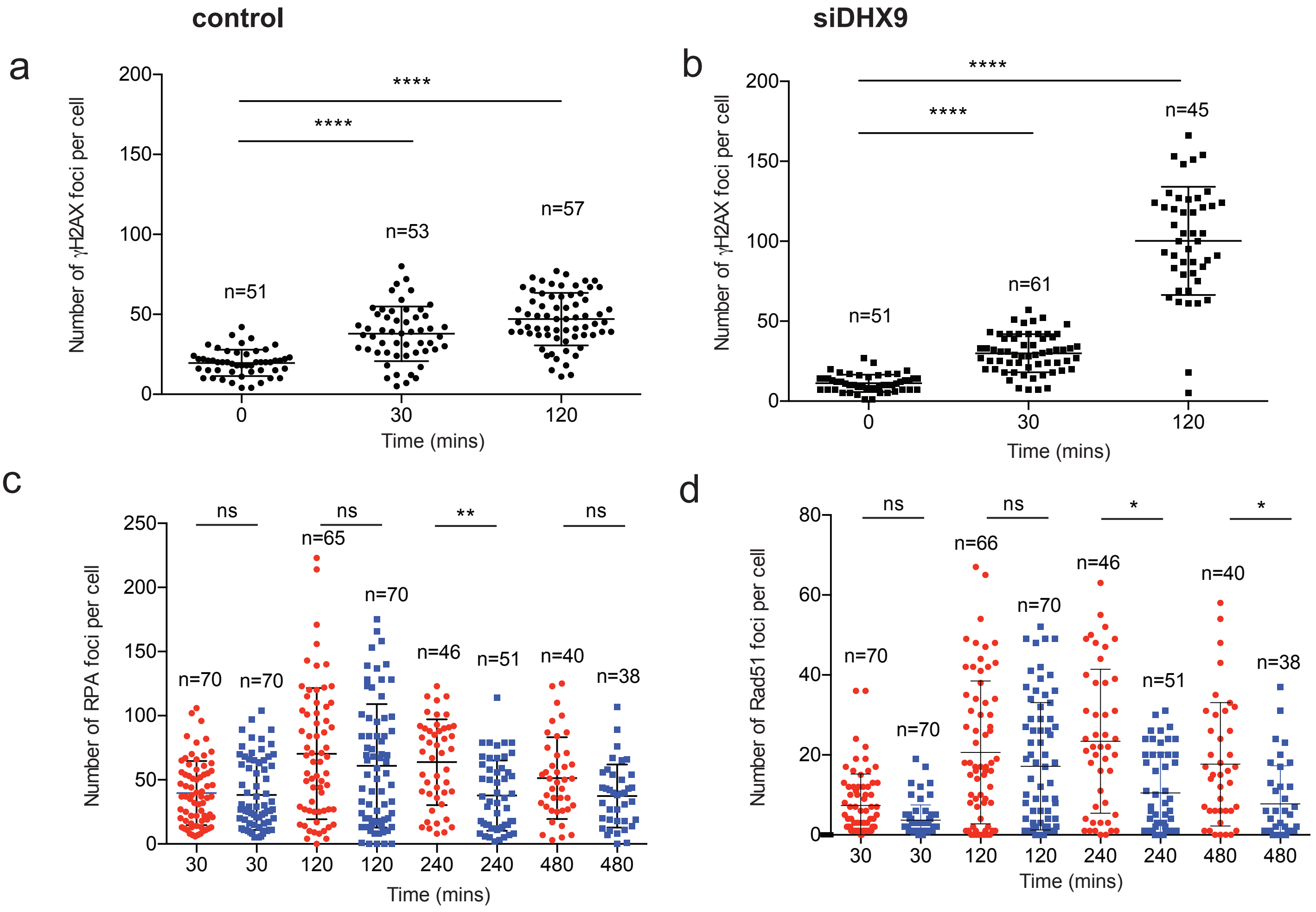


**Supplementary Figure 4.** (**a**) Number of γH2AX foci per cell after treatment of siControl cells with IR (**b**) Number of γH2AX foci per cell after treatment of siDHX9 cells with IR (**c**). Number of RPA foci per cell after treatment with IR in siControl (red) and siDHX9 cells (blue) (**d**). Number of RAD51 foci per cell after treatment of siControl (red) and siDHX9 (blue) cells with IR. For all graphs, quantification of n cells (as indicated) from 3 pooled biologically independent experiments were performed. Statistical significance was determined using one way ANOVA with Tukey's post hoc test (*$p < 0.1$, **$p < 0.01$, ***$p < 0.001$, ****$p < 0.0001$). Mean and error bars indicating one standard deviation are also indicated.

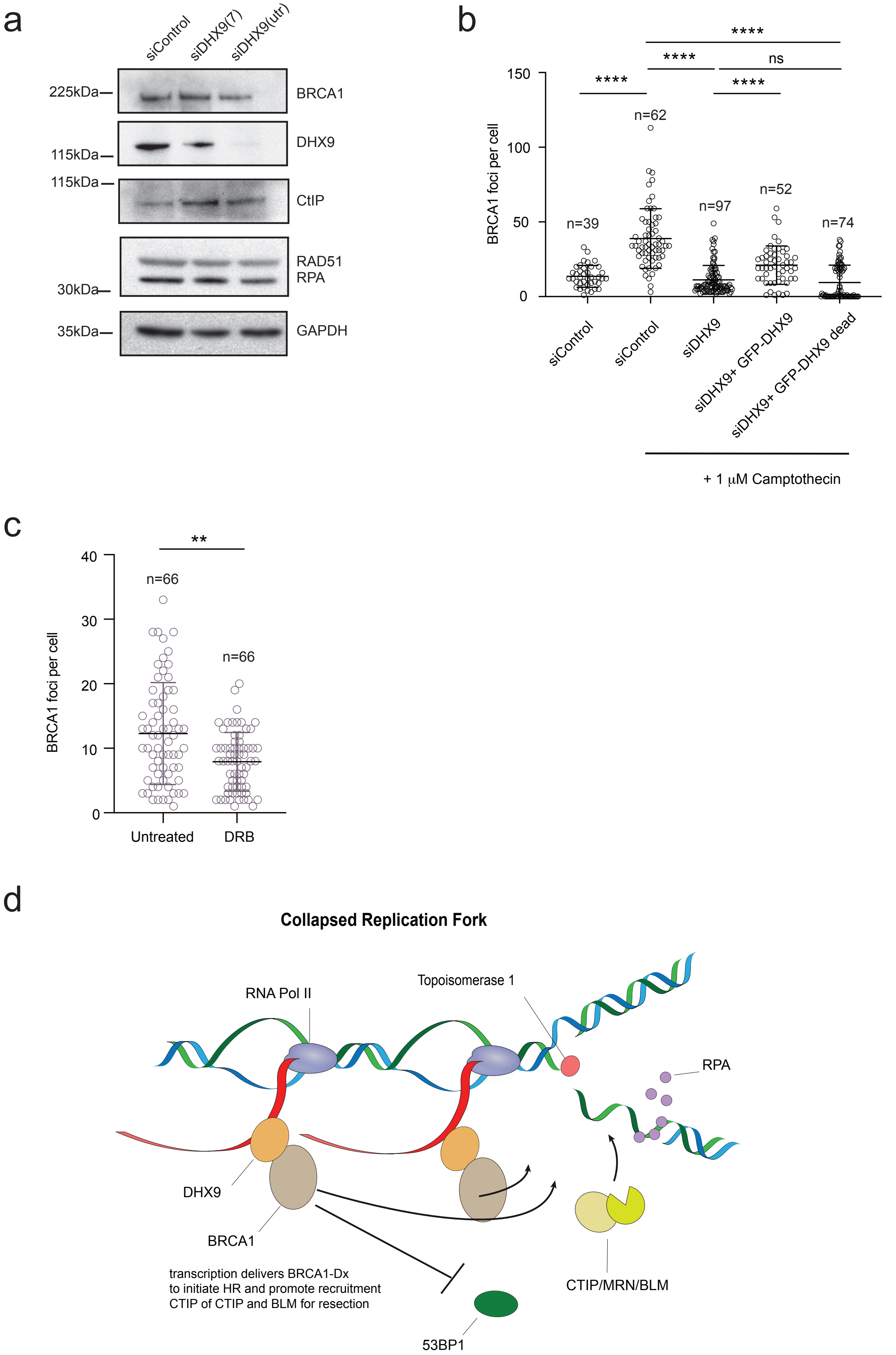
a
siControl
siDHX9(7)
siDHX9(utr)
225kDa
115kDa
115kDa
30kDa
35kDa
BRCA1
DHX9
CtIP
RAD51
RPA
GAPDH
b
150
100
50
0
BRCA1 foci per cell
****
****
****
ns
****
n=39
n=62
n=97
n=52
n=74
siControl
siControl
siDHX9
siDHX9+ GFP-DHX9
siDHX9+ GFP-DHX9 dead
+ 1 μM Camptothecin
c
40
30
20
10
0
BRCA1 foci per cell
**
n=66
n=66
Untreated
DRB
d
Collapsed Replication Fork
RNA Pol II
Topoisomerase 1
RPA
DHX9
BRCA1
transcription delivers BRCA1-Dx
to initiate HR and promote recruitment
CTIP of CTIP and BLM for resection
CTIP/MRN/BLM
53BP1

**Supplementary Fig 5.** (**a**) Western blot showing that knockdown of DHX9 with two different siRNAs does not affect the levels of key HR proteins BRCA1, CTIP, RPA and RAD51. GAPDH is shown to confirm equal loading of the gel. We note that knockdown of DHX9 using siDHX9 (7) in this experiment is incomplete. (**b**) Quantification of fluorescence images detecting the formation of BRCA1 foci after treatment of cells with 1μM camptothecin as described in Fig.6. Graph shows that recruitment of BRCA1 to sites of DNA damage is restored in cells depleted of DHX9 using siDHX9 (utr) by expression of wild type but not helicase dead DHX9 mutant (indicated). (**c**) Graph of data from fluorescence imaging showing that the formation of BRCA1 foci is diminished in cells inhibited for transcription using the RNA Pol II inhibitor DRB. Quantification of *n* cells (as indicated) from 3 pooled biologically independent experiments were performed in (b) and (c). Statistical significance was determined using one way ANOVA with Tukey's post hoc test (****$p < 0.0001$,***$p < 0.01$, ns = not significant). Mean and error bars indicating one standard deviation are also indicated. (**d**) Model showing the repair of a collapsed replication fork caused by camptothecin inhibition of Topo1. In regions of transcription RNA Pol II delivers BRCA1-DX (BRCA1 and DHX9) to DSB where it inhibits 53BP1 and facilitates the recruitment of CTIP/MRE11/BLM to initiate DNA resection and HR mediated repair. Single-stranded DNA generated by resection is bound initially by RPA and subsequently by the RAD51 recombinase. Source data are provided as a Source Data file.

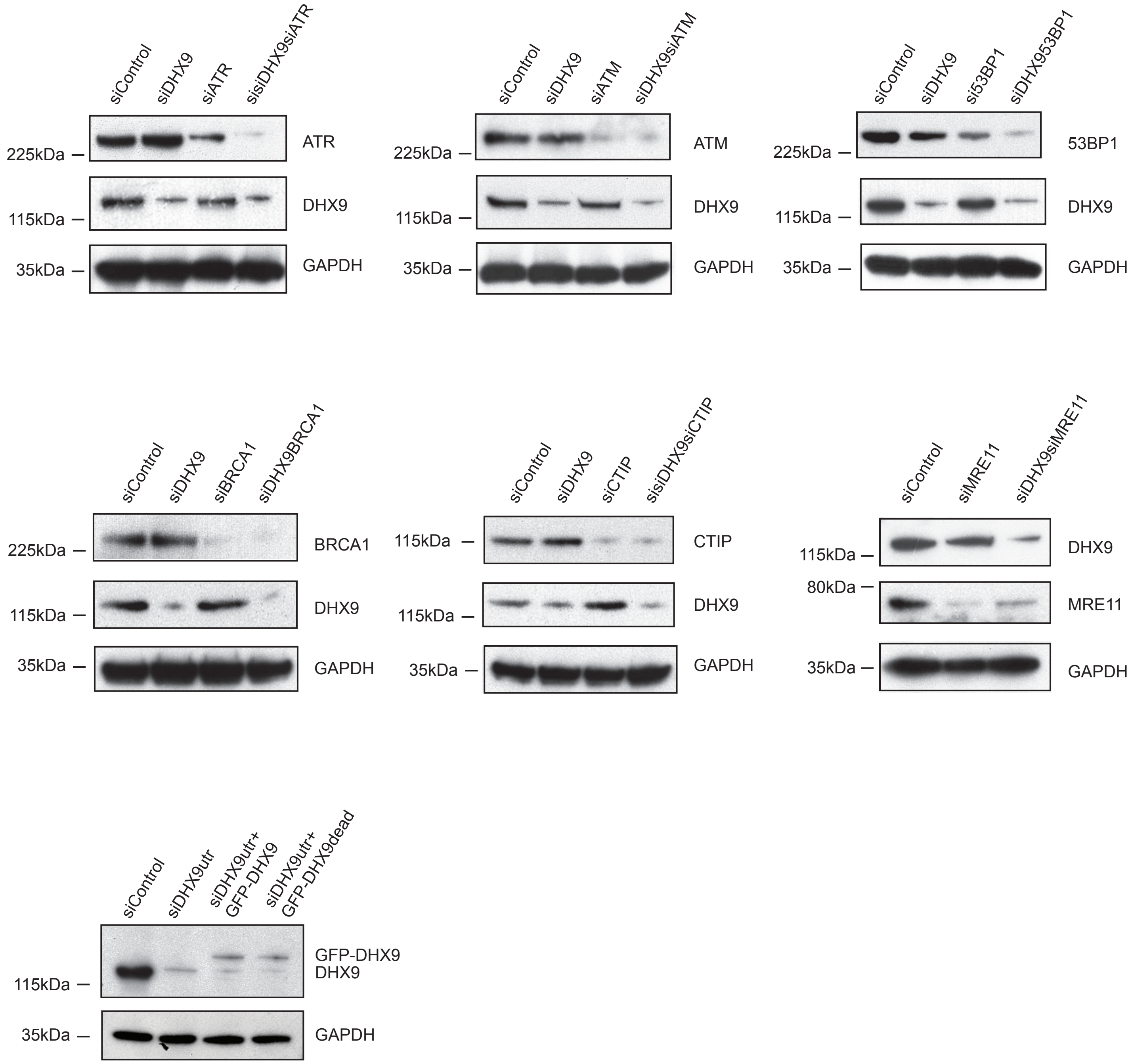


**Supplementary Figure 6**- Western blots showing depletion of indicated proteins using siRNA.
