# Supplementary material for: DHX9-dependent recruitment of BRCA1 to RNA promotes DNA end resection in homologous recombination
Source: Nat Commun. 2021 Jul 5;12:4126. doi: 10.1038/s41467-021-24341-z (PMC8257769; doi:10.1038/s41467-021-24341-z)
Supplement: Supplementary file 3 — Reporting Summary [file 41467_2021_24341_MOESM3_ESM.pdf]

nature research

Corresponding author(s): Kevin Hiom

Last updated by author(s): Apr 30, 2021

Reporting Summary

Nature Research wishes to improve the reproducibility of the work that we publish. This form provides structure for consistency and transparency in reporting. For further information on Nature Research policies, see our Editorial Policies and the Editorial Policy Checklist.

Statistics

For all statistical analyses, confirm that the following items are present in the figure legend, table legend, main text, or Methods section.

n/a	Confirmed	
☐	☒	The exact sample size (*n*) for each experimental group/condition, given as a discrete number and unit of measurement
☒	☐	A statement on whether measurements were taken from distinct samples or whether the same sample was measured repeatedly
☐	☒	The statistical test(s) used AND whether they are one- or two-sided<br>*Only common tests should be described solely by name; describe more complex techniques in the Methods section.*
☒	☐	A description of all covariates tested
☐	☒	A description of any assumptions or corrections, such as tests of normality and adjustment for multiple comparisons
☐	☒	A full description of the statistical parameters including central tendency (e.g. means) or other basic estimates (e.g. regression coefficient) AND variation (e.g. standard deviation) or associated estimates of uncertainty (e.g. confidence intervals)
☐	☒	For null hypothesis testing, the test statistic (e.g. *F*, *t*, *r*) with confidence intervals, effect sizes, degrees of freedom and *P* value noted<br>*Give P values as exact values whenever suitable.*
☒	☐	For Bayesian analysis, information on the choice of priors and Markov chain Monte Carlo settings
☒	☐	For hierarchical and complex designs, identification of the appropriate level for tests and full reporting of outcomes
☒	☐	Estimates of effect sizes (e.g. Cohen's *d*, Pearson's *r*), indicating how they were calculated

Our web collection on statistics for biologists contains articles on many of the points above.

Software and code

Policy information about availability of computer code

Data collection	All microscope data: acquisition software was using the DeltaVision SoftWoRx 6.5.2 software suite (GE Healthcare).
Data analysis	Colocalization between two proteins was assessed in single-cell regions using SoftWoRx (version 5.5.0, release 6). A rectangular selection was defined for each cell in deconvolved images, and the Pearson coefficient of correlation was calculated for the volume. Means values were established as significant using one-way ANOVA test<br>Nuclear foci were quantified using an ImageJ macro (supplemental). Nuclei were automatically detected in the DAPI channel using the Default ImageJ auto-thresholding method, and foci in the red and green channels were counted using ImageJ's "Find Maxima" feature with a user-defined Prominence above background.

For manuscripts utilizing custom algorithms or software that are central to the research but not yet described in published literature, software must be made available to editors and reviewers. We strongly encourage code deposition in a community repository (e.g. GitHub). See the Nature Research guidelines for submitting code & software for further information.

Data

Policy information about availability of data

All manuscripts must include a data availability statement. This statement should provide the following information, where applicable:

- Accession codes, unique identifiers, or web links for publicly available datasets
- A list of figures that have associated raw data
- A description of any restrictions on data availability

All data is available on request. Raw data for graphs, tables and gels is included as source data with the manuscript published by the journal.

Field-specific reporting

Please select the one below that is the best fit for your research. If you are not sure, read the appropriate sections before making your selection.

☒ Life sciences ☐ Behavioural & social sciences ☐ Ecological, evolutionary & environmental sciences

For a reference copy of the document with all sections, see nature.com/documents/nr-reporting-summary-flat.pdf

Life sciences study design

All studies must disclose on these points even when the disclosure is negative.

Sample size	Sample size was sufficient for robust statistical significance for a given experimental procedure (e.g. number of cells per slide). All clonogenic survival experiments were undertaken with three independent biological replicates. Analysis of cell images were performed with sufficient cell numbers and over n=3 independent biological replicates to establish a high degree of statistical significance.
Data exclusions	no data excluded
Replication	Experiments were performed multiple times (three or more) with multiple biological replicates. All replication was successful.
Randomization	Where possible all experiments were performed blind. For the analysis of images acquisition of cells was performed automatically in a non-biased way by software (described above) and all images quantified without selection using image J software.
Blinding	Data collection of all images was blinded to the experimentrr. Slides of cells were scanned automatically by microscope software.

Reporting for specific materials, systems and methods

We require information from authors about some types of materials, experimental systems and methods used in many studies. Here, indicate whether each material, system or method listed is relevant to your study. If you are not sure if a list item applies to your research, read the appropriate section before selecting a response.

Materials & experimental systems

n/a	Involved in the study	
☐	☒	Antibodies
☐	☒	Eukaryotic cell lines
☒	☐	Palaeontology and archaeology
☒	☐	Animals and other organisms
☒	☐	Human research participants
☒	☐	Clinical data
☒	☐	Dual use research of concern

Methods

n/a	Involved in the study	
☒	☐	ChIP-seq
☐	☒	Flow cytometry
☒	☐	MRI-based neuroimaging

Antibodies

Antibodies used	Primary antibodies used in this study and concentrations used for immunoprecipiatation (IP), immunofluorescence (IF) and Western blot (WB) are indicated. Rabbit polyclonal anti-RNA Helicase A (ab26271, Abcam, for IP 5 µg/ml, IF 1:1000 and WB 1:2000 dilution), mouse monoclonal anti-BRCA1 antibody (OP92, Ab-1; Calbiochem, for IPs 10[illegible]g/ml, IF 1:250 and WB 1:200 dilution), mouse anti-RPA32 (RPA2 Ab #2; Calbiochem, IF 1:500, WB 1:1000 and for FACS 1:100 dilution) , rabbit polyclonal anti-RNA-Polymerase II (N-20; Santa Cruz Biotechnology, IP 4 [illegible]g/ml and WB 1:1000 dilution), mouse monoclonal anti-GAPDH antibody (GT239; GeneTex, WB 1:1000 dilution), mouse monoclonal anti-phospho-Histone H2A.X (clone JBW301; EMD Millipore, IF 1:250 dilution and WB 1:500 dilution), rabbit anti-phospho-Histone H2A.X (ab81299, Abcam, IF 1:1000 dilution), rabbit polyclonal anti-Rad51 antibody (H-92; Santa Cruz Biotechnology, IF 1:250 dilution), rabbit polyclonal anti-53BP1 Antibody (NB100-904; Novus Biologicals, IF 1:500, WB 1:2000), rabbit polyclonal anti-53BP1 Antibody (A300-272A, Universal biologicals, IF 1:1000 dilution), mouse monoclonal Anti-BrdU antibody (B44; BD Biosciences, 1:1000 dilution for IF), Rat monoclonal anti-BrdU antibody (ab6326, Abcam, IF 1:1000 dilution), rabbit polyclonal anti-CTIP antibody (NB100-79810, Novus Biologicals, WB 1:2000, IF 1:500 dilution), rabbit polyclonal anti-MRE 11 antibody (NB100-142, Novus Biologicals, WB 1:1000 dilution), rabbit polyclonal anti-BLM antibody, (PLA0029, Sigma-Aldrich, WB 1:1000 dilution), mouse monoclonal anti-ATR antibody (NB100-308, Novus Biologicals,WB 1:1000 dilution), rabbit polyclonal anti-ATM antibody (NB100-104, Novus Biologicals, WB 1:1000 dilution), rabbit monoclonal anti-Ku80 Antibody (218, Cell Signalling, WB 1:1000 dilution). Secondary antibodies: goat anti-mouse Alexa Fluor 488 and 647 (Molecular Probes) was used at 1:200 dilution for FACS and 1:1000 dilution for IF, goat anti-rabbit Alexa fluor 488, 568 and 647 (Molecular Probes) was used at 1:1000 dilution for IF, goat anti-rat Alexa 488-conjugated secondary antibody (Molecular Probes, IF 1:600 dilution), goat anti-mouse Alexa 594 conjugated secondary antibody (Molecular Probes, IF 1:500 dilution).<br><br>Secondary antibodies for western blot; Peroxidase-AffiniPure Goat Anti-Rabbit IgG (H+L) 111-035-144-JIR (1:5000 dilution) and Mouse IgG antibody (HRP) (GTX213111-01; GeneTex, 1:5000 dilution) were used. IgG controls were Normal Rabbit IgG #2729 (Cell

	Signaling) and normal mouse IgG (sc-2025; Santa Cruz Biotechnology).
Validation	All antibodies were validated by suppliers and have been also validated by prior publication. In addition each antibody was validated by knockdown of specific protein using siRNA with associated knockdown of specific protein signal as shown for example in in Supp Fif 6 and

Eukaryotic cell lines

Policy information about cell lines

Cell line source(s)	Hela, U2OS (ATCC), H1299-dA3-1 (Prof T Ohno. Division of Genome Biology, National Cancer Center Research Institute, Chuo-ku, Tokyo, Japan)
Authentication	Authenticated by supplier and periodically validated according to ANSI/ATCC standard ASN-0002
Mycoplasma contamination	All cell lines routinely tested for mycoplasma infection using Myco alert from lonza Biologics https://bioscience.lonza.com/lonza_bs/GB/en/Cell-analysis/p/000000000000186462/MycoAlert-Mycoplasma-Detection-Kit-%2810-Tests%29
Commonly misidentified lines (See ICLAC register)	none used in this study

Flow Cytometry

Plots

Confirm that:

☒ The axis labels state the marker and fluorochrome used (e.g. CD4-FITC).

☒ The axis scales are clearly visible. Include numbers along axes only for bottom left plot of group (a 'group' is an analysis of identical markers).

☒ All plots are contour plots with outliers or pseudocolor plots.

☒ A numerical value for number of cells or percentage (with statistics) is provided.

Methodology

Sample preparation	Approximately 75,000 cells were plated overnight before siRNA transfections. Cells were then transfected for 24-48 hours and labelled with BrdU. After trypsinization, cells were labelled with anti-BrdU antibody and propidium iodide (PI) for staining and analysis. Quantification of cell cycle events was performed using FITC and PI according to the manufacturer's instructions (BD Biosciences).
Instrument	Fortessa flow cytometer (Beckton Dickinson)
Software	FlowJo v 10.7
Cell population abundance	10,000 events were recorded for each sample.
Gating strategy	FACS analysis was performed gating for FITC-A (SSC) and Propidium iodide (FSC). Gating strategy is depicted in source file. the boundaries for the percentage of cells in each gate represents the relative number of cells in G1, S, and G2/M. BrdU(FITC) staining intensity (DNA synthesis) is measured by on a logarithmic scale on the Y-axis while PI staining on the X-axis measures the doubling of DNA content i.e 2C to 4C. Raw data for percent cells in each gated region for three independent replicates is provided in Supplementary figure.

☒ Tick this box to confirm that a figure exemplifying the gating strategy is provided in the Supplementary Information.
